# The Critical Roles of Methyltransferase‐Like 3 in Atherosclerosis and Arterial Infarction: Breakthroughs in Mechanism Understanding

**DOI:** 10.1111/jcmm.70897

**Published:** 2025-10-16

**Authors:** Ruida Liu, Lei Yang, Dongqiong Xiao

**Affiliations:** ^1^ Department of Pediatrics/Key Laboratory of Birth Defects and Related Diseases of Women and Children (Ministry of Education) West China Second University Hospital, Sichuan University Chengdu China; ^2^ West China School of Medicine Sichuan University Chengdu Sichuan China; ^3^ Department of Emergency/Key Laboratory of Birth Defects and Related Diseases of Women and Children (Ministry of Education) West China Second University Hospital, Sichuan University Chengdu China

**Keywords:** arterial infarction, atherosclerosis, methyltransferase‐like 3, RNA N^6^‐methyladenosine modification

## Abstract

Atherosclerosis (AS) and arterial infarction currently have a significant impact on human health. Due to their complex pathogenesis and lack of effective treatment approaches, patients worldwide experience high mortality rates. Methyltransferase‐like 3 (METTL3)‐mediated RNA N^6^‐modification has garnered considerable attention in elucidating the mechanisms underlying the pathogenesis and treatment failure of AS and arterial infarction. However, few reviews have explored the interrelationship between them. Therefore, this review aims to address this literature gap by evaluating all studies reporting on the involvement of METTL3 in AS and arterial infarction, as well as targeting METTL3 for rescuing these diseases. This review suggests that METTL3 serves as a risk factor, diagnostic biomarker and therapeutic target for AS and arterial infarction. Overall, it provides a comprehensive overview of cutting‐edge research on METTL3 in AS and arterial infarction, serving as a convenient reference for researchers in both basic and translational medicine fields.

**Abbreviations**
AAA: abdominal aortic aneurysms
Ang II: angiotensin
AS: atherosclerosis
AVC: aortic valve calcification
DGCR8: DiGeorge syndrome critical region 8
ECM: extracellular matrix
ECs: endothelial cells
EGFR: epidermal growth factor receptor
FSP1: NADPH‐ferroptosis suppressor protein 1
H/R: hypoxia/reperfusion
HDGF: hepatoma‐derived growth factor
HTZZW: Hua Tuo Zai Zao Wan
IGF2BP1: insulin‐like growth factor 2 mRNA binding protein 1
IRF‐1: interferon regulatory factor‐1
JAK2: Janus kinase 2
KLF4: Krüppel‐like factor 4
m^6^A: N^6^‐methyladenosine
MAPK: mitogen‐activated protein kinase
Matr3: Matrin‐3
METTL3: methyltransferase‐like 3
NF‐κB: nuclear factor‐kappa B
NLRP1: NLR family pyrin domain containing 1
ox‐LDL: oxidised low‐density lipoprotein
PDK1: protein kinase 1
SLC7A11: solute carrier family 7 member 11
SMCs: smooth muscle cells
STAT3: signal transducer and activator of transcription 3
TWIST1: twist‐related protein 1

## 1 Introduction

Atherosclerosis (AS) and arterial infarction are prevalent vascular disorders, currently ranking as the leading cause of mortality worldwide [1, 2]. Their pathogenesis relies on intricate regulatory mechanisms that have been extensively investigated from various perspectives, particularly focusing on the N^6^‐methyladenosine (m^6^A) modification at the RNA level [3].

The m^6^A modification of RNAs is regulated by methyltransferases, demethylases and m^6^A‐reader proteins [4, 5, 6, 7]. Among these, methyltransferase‐like 3 (METTL3) belongs to the group of methyltransferases and serves as the sole catalytic regulator during m^6^A modification on RNAs. However, it still requires support from other members involved in m^6^A modification such as methyltransferases (e.g., METTL14), demethylases (e.g., FTO/ALKBH5) and readers (e.g., N^6^‐methyladenosine RNA binding protein F or YTHDF) [8, 9]. Collectively, these members control the translation and degradation of target RNAs [10]. Research on METTL3‐regulated m^6^A modification has garnered attention due to its indispensability in the pathogenesis and treatment of AS and arterial infarction. Despite extensive studies on the roles of METTL3 in AS and artery infarction, few reviews comprehensively interpret their interrelationship or evaluate METTL3's prospective applications in a clinical setting.

Therefore, we focus specifically on AS and arterial infarction due to their high clinical prevalence, shared pathophysiological mechanisms and the growing body of evidence implicating METTL3 in these processes. Although METTL3 also influences other vascular diseases, the depth of research in AS and arterial infarction provides a cohesive narrative for mechanistic and therapeutic exploration. In this review, our objective is to address the existing literature gap by providing a comprehensive summary and analysis of all studies that have reported on the involvement of METTL3 in AS and arterial infarction. Additionally, we aim to assess the potential of METTL3 as a clinical target for both diagnosing and treating these diseases. Collectively, this review contributes significantly to enhancing our understanding of the mechanisms underlying METTL3‐mediated m^6^A regulation in AS and arterial infarction.

## 2 Methods for Literature Review

Studies included in this review were identified through PubMed and Web of Science databases using the following keywords: ‘METTL3’, ‘m^6^A’, ‘atherosclerosis’, ‘arterial infarction’, ‘aortic aneurysm’, ‘coronary syndrome’, ‘endothelial cell’, ‘macrophage’, ‘smooth muscle cell’. Articles published between January 2010 and December 2023 were considered. Both preclinical and clinical studies were included, with priority given to original research articles demonstrating mechanistic insights. Review articles and non‐English publications were excluded.

## 3 Relationship Between METTL3 and AS

The pathogenesis of AS involves chronic endothelial cell (EC) injury, lipid accumulation, plaque formation, macrophage inflammation response, smooth muscle cell (SMC) proliferation and vascular injury in arterial walls during the progression of cardiovascular disorders [11, 12]. RNA m^6^A modification has been implicated in AS pathogenesis [13, 14, 15]. Transcriptome‐wide analysis of m^6^A methylation profiles in AS mice reveals significant increases in global levels of m^6^A and METTL3 expression within the aorta. These modifications mediate gene regulation within AS‐relevant pathways [14]. Furthermore, Xu et al. [13] observed overexpression of METTL3 in patients with AS compared to non‐AS individuals. Collectively, these findings highlight the potential role of METTL3‐mediated m^6^A modification in the pathogenesis of AS.

### 3.1 METTL3 Functions by Inducing EC Dysfunction

Vascular EC injury and dysfunction can lead to vasoconstriction, oxidative stress, platelet aggregation and smooth muscle proliferation [16]. EC dysfunction has been observed in various arterial diseases, particularly AS and artery infarction [12]. Given that METTL3 regulates the biological function of ECs, its role in vascular diseases has also been emphasised.

**FIGURE 1:**
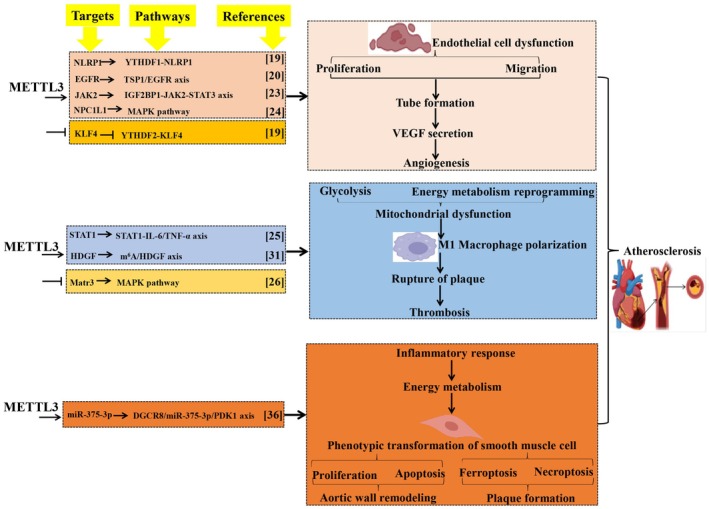
METTL3 contributes to atherosclerosis. METTL3 contributes to the onset and progression of atherosclerosis by regulating vascular endothelial cell dysfunction, macrophage polarisation and phenotypic transformation of smooth muscle cells. This image depicts a comprehensive summary and evaluation of the targets, mechanisms, functions and outcomes of METTL3 in atherosclerosis. Methyltransferase‐like 3 (METTL3). NLR family pyrin domain containing 1 (NLRP1). N^6^‐Methyladenosine RNA binding protein F (YTHDF). Epidermal growth factor receptor (EGFR). Thrombospondin‐1 (TSP‐1). Insulin‐like growth factor 2 mRNA binding protein 1 (IGF2BP1). Janus kinase 2 (JAK2). Signal transducer and activator of transcription (STAT). Extracellular matrix (ECM). Mitogen‐activated protein kinase (MAPK). Krüppel‐like factor 4 (KLF4). Vascular endothelial growth factor (VEGF). Tumour necrosis factor‐α (TNF‐α). Signal transducer and activator of transcription 1 (STAT1). Hepatoma‐derived growth factor (HDGF). N^6^‐methyladenosine (m^6^A). Matrin‐3 (Matr3). DiGeorge syndrome critical region 8 (DGCR8). Protein kinase 1 (PDK1).

AS preferentially occurs at branches and curvatures in the arterial tree where the human umbilical vein EC is exposed to disturbed flow [17]. Disturbed flow increases the inflammatory response and expression of intercellular adhesion molecules in injured EC [18]. Oxidative stress induces EC damage and disturbs blood flow [18]. Chien et al. [19] reported that oxidative stress induces METTL3 upregulation, leading to m^6^A hypermethylation, phosphorylation of nuclear factor‐kappa B (NF‐κB), p65, as well as Ser536, and monocyte adhesion. Consistently, METTL3‐mediated RNA hypermethylation increases NLR family pyrin domain containing 1 (NLRP1) expression and decreases Krüppel‐like factor 4 (KLF4) expression via YTHDF1 and YTHDF2 in different ways. Interestingly, in an in vivo AS model, METTL3 and NLRP1 are upregulated, whereas KLF4 is downregulated. However, METTL3 knockdown results in NLRP3 upregulation and KLF4 downregulation, effectively preventing oxidative stress‐induced AS and disturbing blood flow. This study suggests that the upregulated levels of METTL3 trigger EC dysfunction and vascular injury during AS. Additionally, METTL3 activates the TSP‐1 (an epidermal growth factor receptor (EGFR) ligand)/EGFR axis and helps enhance *EGFR* mRNA stability. EGFR induces EC dysfunction and vascular pathophysiology, ultimately contributing to AS occurrence and progression [20]. Collectively, these findings suggest that METTL3 facilitates endothelial atherogenic progression through m^6^A‐dependent stabilisation of *EGFR* mRNA (Figure 1).

Oxidised low‐density lipoprotein (ox‐LDL) deposition in the vascular endothelium is one main cause of AS, which damages vascular EC and leads to the accumulation of lipids and M1 inflammatory macrophage in the damaged area, thereby forming AS plaques [21, 22]. Research indicates that ox‐LDL triggers EC activation and dysfunction via multiple mechanisms that are likely associated with METTL3. Dong et al. [23] reported that in ox‐LDL‐induced dysregulated EC, METTL3 expression level is high, which operates in concert with insulin‐like growth factor 2 mRNA binding protein 1 (IGF2BP1) to augment Janus kinase 2 (JAK2) expression and activates the JAK2/signal transducer and activator of transcription 3 (STAT3) pathway in EC. Subsequently, the signal triggered by METTL3 is transduced through the JAK2‐STAT3 axis to promote cell proliferation, migration, tube formation, VEGF secretion, angiogenesis in developing embryos and AS progression [23]. The decreased METTL3 levels may confer protection against AS progression. *NPC1L1* is the most significantly downregulated transcript in response to METTL3 knockdown [24]. Moreover, knockdown of NPC1L1 or METTL3‐mediated downregulation of NPC1L1 improves ox‐LDL‐induced dysfunction of human umbilical vein EC in vitro and high‐fat diet‐induced atherosclerotic plaque in vivo, which is associated with the inactivation of the mitogen‐activated protein kinase (MAPK) pathway [24]. METTL3‐mediated *NPC1L1* mRNA hypermethylation facilitates AS progression by regulating the MAPK pathway, and NPC1L1 may be a novel target for the treatment of AS [24] (Figure 1).

### 3.2 METTL3 Induces Inflammatory Macrophage

ox‐LDL exposure significantly amplifies METTL3‐mediated m^6^A modification of mRNA and the inflammatory response in macrophages, and METTL3 affects STAT1 expression and activation [25]. Moreover, ox‐LDL exposure facilitates the interaction between METTL3 and STAT1, promoting STAT1‐mediated transcriptional regulation of inflammatory factor expression in macrophages [25]. Thus, METTL3 is likely to promote ox‐LDL‐induced inflammation by targeting STAT1 in macrophages, making METTL3 a potential therapeutic target for treating AS. In line with this study, Sun et al. [26] reported that ox‐LDL stimulation increases the expression of METTL3 and METTL14 in macrophages, whereas the total m^6^A modification level in macrophages is decreased under ox‐LDL stimulation. Matrin‐3 (Matr3), an RNA binding protein, plays a suppressive role in ox‐LDL‐mediated macrophage inflammatory responses by inactivating the pro‐inflammatory signalling and the MAPK, regulating m^6^A‐mediated mRNA decay and the METTL3‐METTL14 complex formation [26]. Matr3 expression is decreased in the ox‐LDL‐stimulated macrophages and the peripheral blood‐derived monocytes from patients with AS. Overexpression of Matr3 significantly alleviates AS development in vivo [26]. Therefore, the role of Matr3 in macrophage inflammatory responses supplies a deep understanding of the relationship between m^6^A modification and AS (Figure 1).

Macrophage polarisation participates in the initiation and progression of AS, which is regulated by cell energy metabolism reprogramming [27]. Specifically, M1 macrophages obtain energy via glycolysis, whereas M2 macrophages utilise mitochondrial oxidative phosphorylation for energy generation [28]. The energy metabolism of macrophages in the plaque can be affected by cytokines, oxidised lipids and hypoxia, leading to the sudden rupture of plaque and subsequent thrombosis, which is the leading cause of AS death and disability [29]. Glycolysis is increased significantly in atherosclerotic plaques in the carotid arteries, suggesting that M1 macrophages are the dominant type in AS [30]. Hepatoma‐derived growth factor (HDGF) expression is elevated in the aortas of patients with AS and AS models of mice, as well as in M1 macrophages, which are associated with the energy metabolism reprogramming of M1 macrophages [31]. The specific deficiency of HDGF in macrophages results in a significant reduction of plaque area, inflammation and M1 macrophage content in the mouse model of AS [31]. Notably, HDGF is a risk factor for AS by regulating the energy metabolism reprogramming of M1 macrophages. Zheng et al. [31] indicated that the high level of METTL3‐mediated m^6^A methylation on *HDGF* mRNA enhances *HDGF* mRNA stability and increases HDGF expression in M1 macrophages, which induces AS by regulating M1 macrophage polarisation through energy metabolism reprogramming. Importantly, deletion of HDGF weakens inflammation, glycolysis and lipid accumulation in M1 macrophages and rescues mitochondrial dysfunction [31]. Therefore, METTL3‐mediated m^6^A methylation on *HDGF* promotes AS progression by regulating macrophage polarisation through energy metabolism reprogramming. As this study highlighted that METTL3 is the upstream key regulator for macrophage polarisation, targeting METTL3 might be a potential therapy for AS. The traditional Chinese medicine compound formulation Hua Tuo Zai Zao Wan (HTZZW) effectively promotes blood circulation and eliminates blood stasis [32]. Consumption of HTZZW significantly decreases the proportion of M1 macrophages in the peripheral blood [32]. Yu et al. [32] found that HTZZW not only inhibits the expression of METTL14, METTL3, and the overall RNA methylation level but it also decreases the m^6^A modification level on specific sites of *NF‐κB* mRNA. Therefore, HTZZW alleviates AS progression by regulating the expression of METTL14 and METTL3 in macrophages, eliminating m^6^A modification on *NF‐κB* mRNA, influencing *NF‐κB* stability, and ultimately resulting in the deactivation of inflammatory macrophages (Figure 1).

### 3.3 METTL3 Functions by Inducing the Abnormal Phenotype of SMC

The abnormal proliferation and migration of SMC are among the most important pathological contributors to AS development [33]. METTL3 is upregulated in the arteries with AS lesions and the proliferation and migration model of human coronary artery SMC [34]. Inhibition of METTL3 weakens the pathological process of human coronary artery SMC in AS by regulating protein synthesis and energy metabolism [34]. These results reveal a new m^6^A epigenetic method to regulate the progress of AS, which suggests approaches for the prevention and treatment of coronary artery SMC‐induced AS by targeting METTL3. Additionally, vascular SMC is a crucial cell type in AS plaques, which possess extensive plasticity and modulate their phenotypes according to the plaque microenvironment [35]. The phenotypic transformation of vascular SMC is a marker of vascular remodelling that leads to AS [35]. Chen et al. [36] reported that silencing METTL3 limits the phenotypic transformation of vascular SMC induced by ox‐LDL. METTL3 downregulation impairs the binding of DiGeorge syndrome critical region 8 (DGCR8) to pri‐miR‐375 and limits miR‐375‐3p expression, which further targets 3‐phosphoinositide dependent protein kinase 1 (PDK1) transcription, ultimately alleviating AS progression [36]. Therefore, METTL3‐mediated m^6^A modification promotes vascular SMC phenotype transformation to trigger AS plaque formation via the DGCR8/miR‐375‐3p/PDK1 axis, highlighting that targeting METTL3 is therapeutic for vascular‐induced AS (Figure 1).

### 3.4 Crosstalk Between METTL3 and Other RNA Modifications

Emerging evidence suggests that METTL3‐mediated m^6^A modification may interact with other RNA modifications such as m^5^C and ac^4^C, though direct evidence in AS remains limited. For instance, metabolic intermediates like S‐adenosylmethionine serve as methyl donors for both m^6^A and m^5^C modifications, suggesting potential competition under pathological conditions. Future studies should explore whether METTL3 influences or is influenced by other epitranscriptomic regulators in AS progression.

In summary, the abnormal expression of METTL3 functions to promote AS onset and progression by inducing EC dysfunction, macrophage polarisation and SMC phenotype transformation via mediating m^6^A modification on target RNAs.

## 4 Relationship Between METTL3 and Arterial Infarction

AS is closely related to the occurrence of arterial infarction. Arterial infarction, either occurring in the aortic (acute aortic infarction, aortic valve calcification [AVC], abdominal aortic aneurysms [AAA], aortic dissection) or coronary (coronary syndrome), could be induced by the METTL3 dysfunction, which functions by mediating m^6^A modification on different RNAs [37].

### 4.1 METTL3 in Acute Aortic Infarction

**FIGURE 2:**
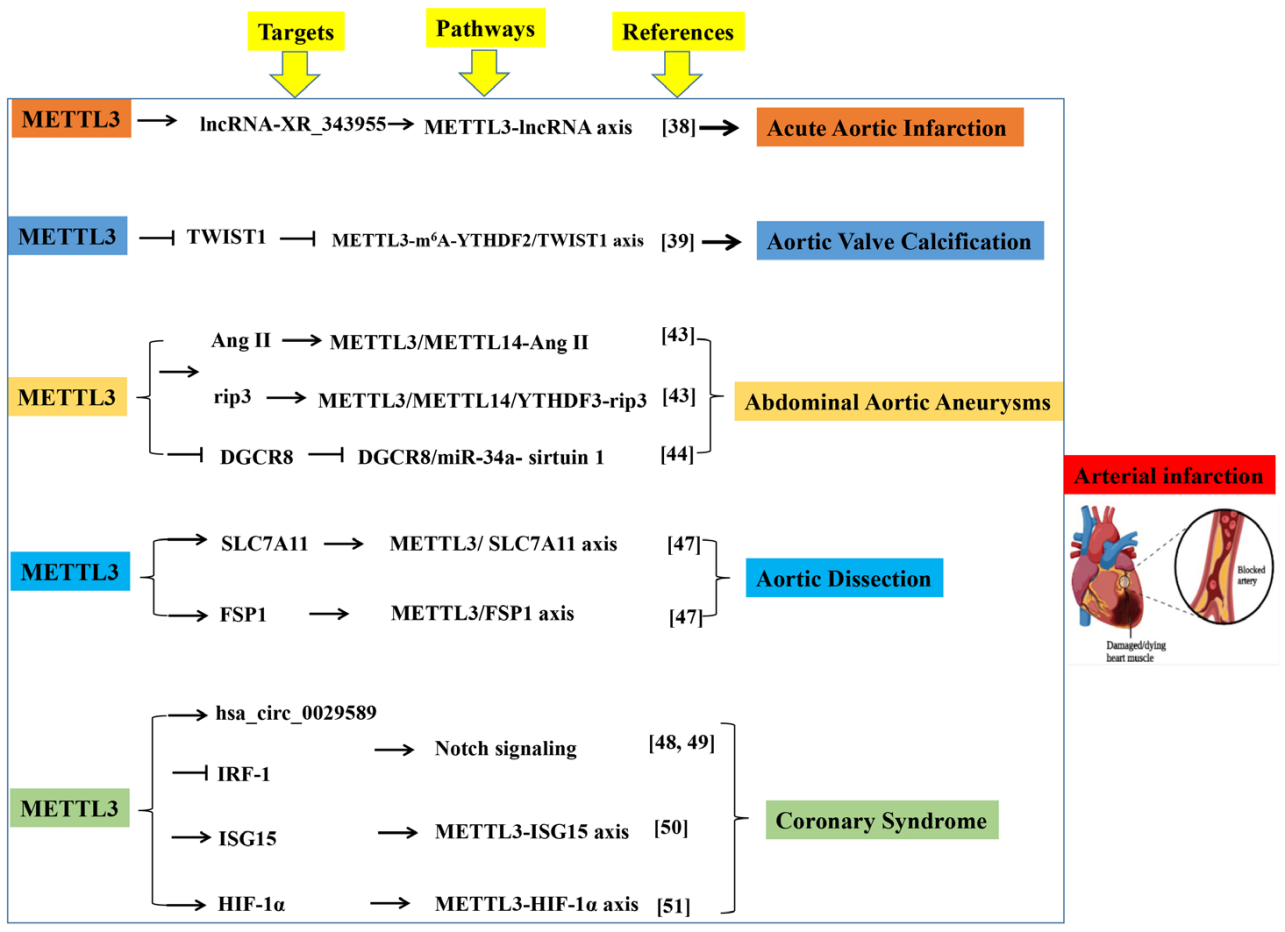
METTL3 contributes to arterial infarction. This image depicts a comprehensive summary and evaluation of the targets, pathway and references of METTL3' role in arterial infarction. Angiotensin (Ang II). Solute carrier family 7 member 11 (SLC7A11). NADPH‐ferroptosis suppressor protein 1 (FSP1). Interferon regulatory factor‐1 (IRF‐1).

In acute aortic infarction induced by EC dysfunction when exposed to lipopolysaccharide, Shen et al. [38] discovered that METTL3 maintains vascular integrity by targeting lncRNA‐XR_343955 and influences the inflammatory response through the cell adhesion molecule pathway. This finding posits METTL3 as a pivotal regulator of vascular integrity during acute inflammatory insults. The specific involvement of lncRNA‐XR_343955 suggests a complex epitranscriptomic network where METTL3 may orchestrate the expression of multiple adhesion molecules (e.g., ICAM‐1, VCAM‐1) through similar mechanisms, ultimately amplifying leukocyte recruitment and vascular damage—a process central to the initiation of acute infarction. Targeting this METTL3‐lncRNA axis could therefore represent a novel strategy to stabilise the vasculature in sepsis‐induced aortic injury (Figure 2, Table 1).

**TABLE 1:** Upregulation of METTL3 contributes to arterial infarctions.

METTL3				
Diseases	Functions	Targets	Mechanisms	References
Acute aortic infarction	EC dysfunction	lncRNA‐XR_343955	Regulates the cell adhesion molecule pathway	[38]
Aortic valve calcification	Osteogenic differentiation of human valvular interstitial cell	*TWIST1*	m^6^A‐YTHDF2‐dependent degradation of TWIST1 mRNA	[39]
Abdominal aortic aneurysms	Necroptosis and inflammation of vascular SMC	*Ang II*	METTL3/METTL14‐Ang II	[40]
	Vascular SMC necroptosis	*rip3*	METTL3‐METTL14/YTHDF3 complex enhances rip3 m^6^A modification and expression	[40]
	Vascular SMC inflammatory response	DGCR8	METTL3 promotes primary miR‐34a processing via DGCR8, leading to SIRT1 downregulation	[41]
Aortic dissection	Ferroptosis of aorta SMC	*SLC7A11/*FSP1	METTL3 mediates mRNA degradation of SLC7A11 and FSP1, promoting ferroptosis	[42]
Coronary Syndromes	Macrophage pyroptosis	*hsa_circ_0029589* *IRF‐1*	METTL3 upregulates hsa_circ_0029589 and inhibits IRF‐1	[43, 44]
	Endothelium‐derived coronary formation	*Notch signalling components*	METTL3 regulates the formation of intramyocardial coronary arteries via modulating Notch signalling	[44]
	Platelet activation & aggregation	*ISG15 mRNA*	METTL3‐mediated m^6^A modification enhances platelet aggregability	[45]
	Cardiomyocyte apoptosis under H/R	*HIF‐1α mRNA*	METTL3 stabilises HIF‐1α mRNA, exacerbating oxidative stress and apoptosis	[46]

*Note:* METTL3 is abnormally upregulated in several arterial infarction diseases, which functions by regulating the functions of EC, valvular interstitial cell and SMC, directly as the oncogene or indirectly by regulating other risk genes.

Abbreviations: DGCR8, DiGeorge syndrome critical region 8; EC, endothelial cell; FSP1, ferroptosis suppressor protein 1; H/R, hypoxia/reperfusion; IRF‐1, interferon regulatory factor 1; SIRT1, Sirtuin 1; SLC7A11, solute carrier family 7 member 11; SMC, smooth muscle cell; VIC, valvular interstitial cell; YTHDF2/3, YTH N^6^‐methyladenosine RNA binding protein F2/F3.

### 4.2 METTL3 in AVC

METTL3 also stimulates the osteogenic differentiation of human valvular interstitial cells by suppressing twist‐related protein 1 (*TWIST1*) via an m^6^A‐YTHDF2‐dependent pathway [39]. This provides novel mechanistic insights into the pivotal role of METTL3 in AVC progression and illuminates new avenues for m^6^A‐targeted diagnostics and therapeutics in AVC (Figure 2, Table 1). The suppression of TWIST1, a key negative regulator of osteogenesis, underscores a fundamental mechanism where METTL3 tips the balance from homeostasis toward pathological calcification. This pathway likely interacts with other pro‐calcific signals in the valve microenvironment, such as BMP/Smad signalling. Future studies should investigate if METTL3 also mediates m^6^A modification of other osteogenic transcripts (e.g., RUNX2, BMP2) in valvular interstitial cells, which would solidify its role as a master regulator of the calcification process and expand the potential for therapeutic intervention.

### 4.3 METTL3 in AAA

AAA is a localised abnormal enlargement of blood vessels, which is usually caused by thinning of the arterial wall due to vascular injury [47]. Chronic vascular wall inflammation, SMC apoptosis and extracellular matrix (ECM) remodelling are critical histopathological features of AAA [47]. Vascular SMC is the primary cell that makes up the aortic wall's midmembrane [48]. An imbalance in vascular SMC proliferation and apoptosis leads to aortic wall remodelling and AAA progression [49]. In angiotensin (Ang II)‐induced AAA, the necroptosis and inflammatory cytokines of vascular SMC are increased [40]. Moreover, the m^6^A modification level and the expression of METTL3/METTL14 are elevated in AAA aortic wall tissues, suggesting that the m^6^A modification is closely associated with Ang II‐induced AAA [40]. Li et al. [40] reported that the METTL3‐METTL14 complex interacts with *rip3* mRNA and SMAD2/3 by depending on YTHDF3 in vascular SMC of Ang II‐induced AAA. The activation of SMAD2/3 in vascular SMC of abdominal aortic wall tissues is stimulated by Ang II, which subsequently promotes METTL3‐METTL14 complex‐mediated m^6^A modification on *rip3* mRNA by promoting the binding between YTHDF3 and *rip3* mRNA, increasing the protein level of rip3, thus contributing to vascular SMC necroptosis, inflammatory response and the AAA pathological process [40]. Interference with METTL3/METTL14 attenuates vascular SMC necroptosis, inflammatory response and AAA progression [40]. Zhong et al. [41] reported that the upregulated level of METTL3 facilitates primary microRNA‐34a maturation to form miR‐34a by targeting DGCR8 during apolipoprotein‐induced AAA formation in vitro and in vivo. Moreover, miR‐34a upregulation remarkably restricts sirtuin 1 expression and exacerbates AAA [41]. These studies suggest that METTL3 plays an important role in AAA formation, providing a novel therapeutic target and diagnostic biomarker for treating this condition (Figure 2, Table 1). The dual role of METTL3 in promoting both vascular SMC necroptosis (via RIP3) and inflammation (via miR‐34a/SIRT1) exemplifies its central position in AAA pathogenesis. This convergence of multiple cell death and inflammatory pathways onto METTL3 suggests that its inhibition could simultaneously mitigate several key histopathological features of AAA. The next critical step is to validate these mechanisms in human AAA tissue samples and to explore the efficacy of cell‐specific Mettl3 knockout or pharmacological inhibition in advanced preclinical models to assess its true therapeutic potential for halting AAA progression.

### 4.4 METTL3 in Aortic Dissection

Loss of SMC is one of the main pathological features of aortic dissection, which induces fragmentation of elastic fibres and degeneration of the aortic wall [50]. Ferroptosis, one programmed cell death type that is driven by the iron‐dependent accumulation of lipid hydroperoxides, has been shown to participate in the loss of SMC during the development of aortic dissection [51]. Solute carrier family 7 member 11 (SLC7A11), NADPH‐ferroptosis suppressor protein 1 (FSP1) and glutathione‐glutathione peroxidase 4 are key regulators of ferroptosis and are downregulated in the aortas of patients with Stanford type A aortic dissection; inhibition of ferroptosis by liproxstatin‐1 largely abrogates β‐aminopropionitrile‐induced aortic dissection in mice [42]. Li et al. [42] found that the expression level of METTL3 in aortic tissues of patients with Stanford type A aortic dissection is higher than that in non‐aortic dissection subjects, which promotes the mRNA degradation of *SLC7A11* and *FSP1* to inhibit their protein expression in primary cultured human aorta SMC. METTL3 accelerates imidazole ketone erastin and cystine deprivation‐induced ferroptosis of human aorta SMC by suppressing SLC7A11 and FSP1 expression, and overexpression of SLC7A11 or FSP1 largely reverses the human aorta SMC ferroptosis caused by METTL3 overexpression [42]. These results indicate that reducing METTL3‐mediated mRNA m^6^A methylation suppresses SMC ferroptosis, as well as inhibiting SMC ferroptosis is a potential strategy to delay the pathological processes of aortic dissection (Figure 2, Table 1). The link between METTL3 and ferroptosis introduces a groundbreaking concept in aortic dissection biology. Given that SLC7A11 is a core component of the cystine/glutamate antiporter system xc−, its suppression by METTL3 would directly disrupt glutathione synthesis and redox homeostasis, priming SMCs for ferroptotic death. This mechanism likely synergises with the well‐established biomechanical stress in the aortic wall. Investigating whether mechanical strain itself can upregulate METTL3 expression, thereby creating a vicious cycle of epitranscriptome‐driven ferroptosis and wall weakening, represents a crucial future direction.

Collectively, these findings establish that METTL3‐driven ferroptosis of vascular SMCs is a critical event in aortic wall weakening and dissection pathogenesis, highlighting a previously unrecognised epitranscriptomic layer regulating vascular cell death.

### 4.5 METTL3 in Coronary Syndrome

Coronary syndromes, including unstable angina and acute myocardial infarction, represent the most prevalent clinical manifestations of arterial infarction, primarily driven by acute thrombosis following atherosclerotic plaque rupture. METTL3's role extends beyond atherogenesis into these acute ischemic events. Guo et al. observed elevated METTL3 expression in macrophages of patients with acute coronary syndrome, where it promotes hsa_circ_0029589 maturation and inhibits interferon regulatory factor‐1 (IRF‐1), thereby activating macrophage pyroptosis and contributing to plaque instability [43]. On the other hand, another study demonstrated that the upregulated level of METTL3 regulates the formation of endocardium‐derived intramyocardial coronary arteries by modulating the Notch signalling [44] (Figure 2, Table 1).

Beyond macrophage pyroptosis, emerging evidence implicates METTL3 in other critical processes of coronary infarction. Recent work suggests METTL3 influences coronary EC senescence and platelet activation. For instance, a 2025 study by Jian et al. [45] showed that METTL3‐mediated m^6^A modification of ISG15 mRNA in platelets enhances their aggregability, providing a novel link between epitranscriptomics and thrombosis in coronary infarction [45]. Furthermore, METTL3 is implicated in regulating the ischemic stress response in cardiomyocytes [46]. Another study demonstrated that METTL3‐mediated m^6^A modification of HIF‐1α mRNA stabilises its transcript, exacerbating oxidative stress and apoptosis in cardiomyocytes subjected to hypoxia/reperfusion (H/R) injury, a key pathological process in myocardial infarction [52, 53, 54, 55]. This multifaceted involvement of METTL3 in endothelial dysfunction, platelet activation, macrophage inflammation and cardiomyocyte death underscores its potential as a central therapeutic target across the spectrum of coronary syndromes (Figure 2, Table 1).

Collectively, METTL3‐mediated m^6^A modification on mRNAs and noncoding RNAs contributes to the dysfunction of EC, loss of SMC and activation of macrophages, which subsequently triggers the occurrence and progression of various artery infarctions, including acute aortic infarction, AVC, AAA, aortic dissection and coronary syndrome.

## 5 Discussion

For the pathogenesis of AS and arterial infarction, EC [56], macrophage [57] and SMC [58] are being regulated by METTL3‐mediated RNA m^6^A modification. Therefore, understanding the mechanism for the pathogenesis and treatment of these diseases (AS, arterial infarction) from the perspective of METTL3‐mediated RNA m^6^A modification has received great attention. This review analyses the roles of METTL3 in AS and arterial infarction, interpreting their interrelationship and evaluating METTL3's prospective applications in the clinical setting.

This review suggests that METTL3 promotes AS onset and progression by inducing EC dysfunction, macrophage polarisation and SMC phenotype transformation via mediating m^6^A modification on different RNAs. The mechanism for METTL3‐mediated m^6^A RNA modification in AS includes the regulation of inflammatory response, oxidative stress, cell proliferation, migration, tube formation, VEGF secretion, angiogenesis, macrophage polarisation, energy metabolism reprogramming, glycolysis, mitochondrial dysfunction and lipids accumulation. Additionally, METTL3‐mediated m^6^A modification on mRNAs and noncoding RNAs contributes to the occurrence and progression of various arterial infarctions, including acute aortic infarction, AVC, AAA, aortic dissection and coronary syndrome by inducing the dysfunction of EC, loss of SMC and activation of macrophage. The underlying mechanism for METTL3 action in artery infarctions includes the regulation of chronic vascular wall inflammation, apoptosis, necroptosis and ferroptosis of SMC, ECM remodelling and macrophage pyroptosis. In conclusion, METTL3 is crucial for regulating vascular diseases, such as AS and arterial infarction, whereas a low level of METTL3 assists in recovery from these conditions. Therefore, targeting METTL3 is a promising biomarker and a therapeutic approach for AS and arterial infarction. While this review focuses on METTL3, it is important to note that other m^6^A regulators (e.g., METTL14, FTO, ALKBH5, YTHDFs) also contribute to vascular pathology. Future reviews may expand on their roles, but the predominant evidence for METTL3 in AS and arterial infarction justifies its central focus herein.

Although our review highlights the promising role of METTL3 as a diagnostic biomarker and therapeutic target, it is crucial to acknowledge that the current evidence is predominantly derived from preclinical models. The transition to clinical application faces several challenges. These include the development of sensitive and specific assays for detecting METTL3 activity or m^6^A levels in human plasma or vascular tissues, the potential off‐target effects of systemic METTL3 inhibition and the heterogeneity of human vascular diseases. Future research must prioritise validating these findings in human cohort studies and developing targeted delivery systems, such as nanoparticle‐based therapies, to selectively modulate METTL3 activity in specific vascular cells before its clinical potential can be fully realised.

However, available publications on the role of METTL3 in arterial infarction are limited, which should be further investigated to bridge the understanding gap of METTL3's role. In addition, although we suggest METTL3 as a potential biomarker and a therapeutic target for AS and arterial infarction, it should be further validated in preclinical studies. Furthermore, this review is primarily focused on interpreting the relationship between METTL3 and the typical vascular cells (ECs, SMCs, macrophages) in AS and artery infarctions. This scope necessarily omits other important contributors to vascular pathology, such as pericytes, fibroblasts and platelets, for which the role of METTL3 remains largely unexplored. Additionally, while we have synthesised findings from available studies, it is important to note common methodological limitations in the field, including the reliance on heterogeneous cellular models of disease (e.g., different ox‐LDL concentrations or stimulation times) and the need for more inducible, cell‐specific knockout models to unequivocally establish METTL3's causal role in vivo. Future studies should therefore not only expand into these neglected cell types but also aim to standardise experimental approaches to improve reproducibility and translational relevance.

In summary, this review provides a comprehensive and updated elucidation of the relationship between METTL3 and AS and arterial infarction, providing a convenient reference for researchers in both basic and translational medicine fields.

## Author Contributions

**Ruida Liu:** conceptualization (equal), formal analysis (equal), supervision (equal), writing – original draft (equal). **Lei Yang:** conceptualization (equal), formal analysis (equal), supervision (equal), writing – original draft (equal). **Dongqiong Xiao:** conceptualization (lead), funding acquisition (lead), supervision (lead), writing – original draft (supporting), writing – review and editing (lead).

## Ethics Statement

The authors have nothing to report.

## Consent

The authors have nothing to report.

## Conflicts of Interest

The authors declare no conflicts of interest.

## Data Availability

No new data were generated or analysed in support of this study.
